# Supplementary material for: Chromothripsis during telomere crisis is independent of NHEJ, and consistent with a replicative origin
Source: Genome Res. 2019 May;29(5):737–49. doi: 10.1101/gr.240705.118 (PMC6499312; doi:10.1101/gr.240705.118)
Supplement: Supplemental Material [file supp_gr.240705.118_Supplemental_file_1.zip › contigs/annotated_contigs/DB49/contig.2.DB49_length_739_mean_cov_5.23951285521.docx]

**DB49_length_739_mean_cov_5.23951285521**

```
TTTTTTTTTTTTTTTTTTTTTTTTTTTTTTTTTTTTTTTTTTTTTTTTTTTTTTTTTTTTTTTTTTTTTTTTT|TTTTTTTTTTTTTTT
 >chr7:107410604-107410739 + E=1e-38 p=0e+00                              >chr8:11880026
TTTTTTTTTTTTGAGACAGAGTCTTGCTCTGT|TGCCTGGGCTGTAGTGCAGTGGCACGATCCGGTTCACTGCAAGATCCGCCTCCTGG
1-118800927 + E=0e+00
GTTCACGTCATTCTCCTGCCTCAGCCTCCCAAGTAGCTGGGACTAAAAGTGCCCGCCACCACGCCCAGTTAATTTTTTGTATTTTTAGT

AGAGACAGGGTTTCACTGTGTTAGCCAGGATGGTCTTGATCTCCTGACCTCGTGATTCGCCCGCCTCAGCCTCCCGAAGTGCTTGGATT

ACAGACAAGTTCACTGATTCTTTAGTCTGCTACATTCAGTCTGTTGTTAAACCCATCAAACAAATTATTTCTGATATTTCTATAGAATT

TCCATTTAGGTCTTTTCTATCTTTTCATGTTTCTGCTTAAACTCCTCAGCTTTTCAATAATGTTGTGCATTGTTCTCACTAGATTATTT

AACATATTTATAACAATTATCTTAAAGTTATTTTCTGCTAATTCCAACAGTTGGGTTGTATGTGGATCTCCTCTATTAATTTTTTCCTC

TTTATCATAGGTATATTTTTCTGTTTCTTTGTATGTCTTGTAATTTTGTATTATATACTAGAAATTGTGTTTAAAAATGATAGTTATGA

CTACAGTATTTCTGTGCTCAGAAAATATC
```
